# Addressing Youth Mental Health Through Schools and Primary Care Clinics Using the Connected for Wellness Mobile App: Protocol for a Stepped-Wedge Trial

**DOI:** 10.2196/73721

**Published:** 2025-08-26

**Authors:** Lisa R Fortuna, Michelle V Porche, Martha Shumway, Roya Ijadi-Maghsoodi, Hilary Aralis, Johanna B Folk, Marina Tolou-Shams, Greg Barish, Juan Carlos Gonzalez, Sheryl Kataoka

**Affiliations:** 1 Psychiatry and Neurosciences School of Medicine University of California, Riverside Riverside, CA United States; 2 Internal Medicine School of Medicine University of California, Riverside Riverside, CA United States; 3 Psychiatry and Behavioral Sciences School of Medicine University of California, San Francisco San Francisco, CA United States; 4 Psychiatry and Biobehavioral Sciences School of Medicine University of California, Los Angeles Los Angeles, CA United States; 5 Public Health Sciences School of Medicine University of California, Davis Davis, CA United States; 6 Allyance Logic Danville, CA United States

**Keywords:** mobile app, co-design, youth mental health, participatory informatics, stepped-wedge design, mental health disparities, wellness, prevention, primary care, school mental health

## Abstract

**Background:**

Youth in the United States are experiencing rising rates of anxiety, depression, and other mental health challenges, yet many remain underserved due to systemic barriers such as poverty, limited access to care, and shortages in the mental health workforce. Schools and primary care clinics are trusted, community-based settings that offer strategic opportunities for early identification, prevention, and intervention. As part of a public health approach, integrating digital mental health tools into these settings can support broad access, enhance mental health literacy, and promote help-seeking behaviors among all students. When co-designed with youth and embedded within existing care systems, these tools offer a scalable, proactive solution to support well-being across diverse school-aged populations.

**Objective:**

This protocol describes the design and implementation of a stepped-wedge clinical trial to evaluate the Connected for Wellness mobile app, a youth- and caregiver-facing digital mental health intervention. Developed using participatory informatics and human-centered design principles, the app provides culturally relevant content in English and Spanish. Features include self-guided wellness activities, well-being screeners, psychoeducational videos, and localized service directories. Machine learning algorithms personalize content recommendations based on user inputs and behavioral patterns.

**Methods:**

This trial uses a stepped-wedge cluster randomized design across 20 community-based sites (10 high schools and 10 primary care clinics) in 2 counties in California. These counties were selected for their high proportions of underserved youth populations. All youth aged 13 to 22 years and their caregivers will be invited to access the app. Sites are randomized into 2 implementation waves. The app is introduced site-wide as a universal public health intervention supported by on-site navigators and peer ambassadors. The primary outcomes are derived from a cascade-of-care framework, including identification of mental health need, referral to services, initiation of services, and engagement (defined as ≥3 treatment visits). Data will be collected via anonymous in-app analytics, monthly World Health Organization–Five Well-Being Index assessments, and deidentified electronic health and administrative records. Generalized linear mixed models will be used to evaluate differences in cascade outcomes between pre- and postimplementation phases while accounting for clustering and site-level variability.

**Results:**

As of May 2025, the mobile app has been finalized, institutional review board approvals have been secured, and all study sites have been recruited. Participant recruitment is projected to begin in August 2025. Data collection and initial analyses will begin in early 2026, with preliminary findings expected by October 2026.

**Conclusions:**

This study tests a novel digital health intervention integrated into trusted care systems. If effective, the Connected for Wellness mobile app may serve as a scalable strategy to reduce disparities in mental health care access and engagement for youth across the United States.

**Trial Registration:**

ClinicalTrials.gov NCT06122688; https://clinicaltrials.gov/study/NCT06122688

**International Registered Report Identifier (IRRID):**

PRR1-10.2196/73721

## Introduction

### Background

Could a mobile mental health app designed for youth and caregivers help address mental health service barriers? For decades, research has documented the ongoing and persistent inequities in mental health care access for many populations, including both rural and urban [1-3]. Innovative aspects of the clinical trial protocol reported in this paper include using a mobile app codeveloped with youth and families through participatory informatics to drive technological innovation considering youth and caregiver priorities regarding customizable features and in-app resources.

Our previous work supports the hypothesis that mobile apps and other digital health interventions are effective for delivering mental health and prevention services provided that they meet the needs and preferences of end users [4]. These interventions can be successfully implemented within child-, adolescent-, and family-serving systems of care. Unlike clinicians’ time, technology offers a nonconsumable strategy (ie, available asynchronous content vs scheduled therapist time) for improving mental health care access [5]. The United States has witnessed an increasing need for youth mental health services, especially for stress-related challenges [6,7] and populations disproportionately affected [8]. National data reveal that, after controlling for sociodemographics, severity of symptoms, income, and health insurance, Black and Latino youth with similar needs as White youth were almost half as likely to receive mental health services [3].

Barriers to care exist across multiple levels, including cultural, community, family, and systemic factors. For instance, Latino caregivers may perceive children’s behavioral challenges as issues to address within the family, struggle with the lack of linguistically and culturally appropriate services, or experience stigma and perceptions of mental health needs that differ from those of mental health care providers [9]. Rural communities of all backgrounds are also underserved. These systematic factors contribute to reduced use of mental health services even in high-risk populations, underscoring the need for focused interventions that can both help mental health symptoms and risks and promote and facilitate engagement with services [10].

Mobile app interventions, integrated into trusted community settings such as schools and primary care clinics, offer scalable solutions to improve access to youth mental health care. To be effective and acceptable, apps need to include features and content that are designed to be engaging for and address the needs of diverse groups [11]. Despite the proliferation of digital health interventions for youth across a range of delivery methods (eg, websites, apps, games, robots, and virtual reality) [12], few are co-designed with the intended users in mind [13]. Furthermore, the methods used for youth and caregiver collaboration in the design process vary widely [14]. This is unfortunate as studies have demonstrated high levels of acceptability among youth for engaging with mobile health app interventions [15]. These tools can be important nonconsumable and scalable approaches for delivering mental health prevention and wellness tools [16]. When mobile mental health apps are used to expand access to mental health education and prevention resources, youth may be more likely to initiate and engage in help-seeking behaviors, and families may report greater satisfaction with care and perceive services as more appropriate once they engage [17].

Accessibility to mobile apps is another important consideration [18,19]. Use of cross-platform frameworks such as React Native [20] can ensure that an app is easily available to a wide community of users while retaining a single codebase for agile development. Mobile apps can also be integrated effectively with school-based and primary care services. Schools and primary care clinics are critical health care and mental health service providers [21], especially for underresourced populations [22]. Pediatric primary care is widely recognized as an important setting for identifying and managing youth mental health needs given that most youth make at least one medical visit annually [23]. In addition, access to mental health supports has often been expanded through educational settings [24], including the adoption of whole-school, multitiered systems of support that provide school-wide preventive interventions and mental health services through school-based health clinics and programs [25].

### Overview of the Connected for Wellness Mobile App

In an effort to address the barriers to care for youth, a co-designed mobile app called Connected for Wellness was created with adolescents and their caregivers. The development of the Connected for Wellness mobile app and its predecessor, Connectd for Schools [26], is grounded in participatory informatics approaches. *Participatory informatics* [27] is an approach to technology codevelopment that combines best practices from community-partnered participatory research [28] and end user–centered participation in technology design [29]. This approach addresses community health priorities by including intended users as equitable partners throughout the design process. The codevelopment of the Connected for Wellness mobile app with youth and caregivers was led by the scientific team that includes diverse, multilingual research staff, child and adolescent psychiatrists, psychologists, technology development experts, and student researchers. The content includes engaging videos created by and representing diverse youth and caregivers in English and Spanish (details on the codevelopment process and protocols are reported elsewhere). In its final form, Connected for Wellness has four key components: (1) mental health and wellness education, (2) self-assessment tools, (3) emotion regulation and coping strategies, and (4) site-specific and community resources. The home page features a Get Help Now button that directly connects the user to 911 for those who need immediate assistance. The community resources include an embedded link to FindHelp, which is searchable by zip code. In addition to listings of financial assistance and free and reduced-cost food, housing, and health care, FindHelp also features helplines for specific needs such as domestic violence assistance and for communities that are particularly underserved. Given our priority to ensure that the app is widely accessible, we also developed a PDF document that provides all app content for anyone who is using a screen reader or prefers reading along.

The app includes optional notifications, inspirational messages, and interactive components to promote ongoing engagement. In addition, the co-design process led to the development of an app that prioritizes user confidentiality and safety. The app allows for anonymous use, does not include 2-way communication, and avoids open-text response fields, minimizing the risk of exposing sensitive personal information and ensuring that users can engage with the content privately and safely. We collaborated with both English- and Spanish-speaking communities to develop Connected for Wellness content in both languages. Table 1 outlines the app’s features and pathways and includes images of the app’s user interface.

To enhance customization and interactivity for both youth and caregivers, the Connected for Wellness mobile app uses collaborative filtering techniques [26] to intelligently recommend wellness skills and supportive resources to study participants. One approach, a content-based (*item-item*) method, automatically suggests skills based on previously viewed skills and the metadata associated with them. For example, the app clusters content related to similar topics—such as sleep health or emotion regulation—by analyzing descriptions, titles, and other metadata, allowing it to recommend new wellness skills that are semantically similar to those the user has already explored.

**Figure 1:**
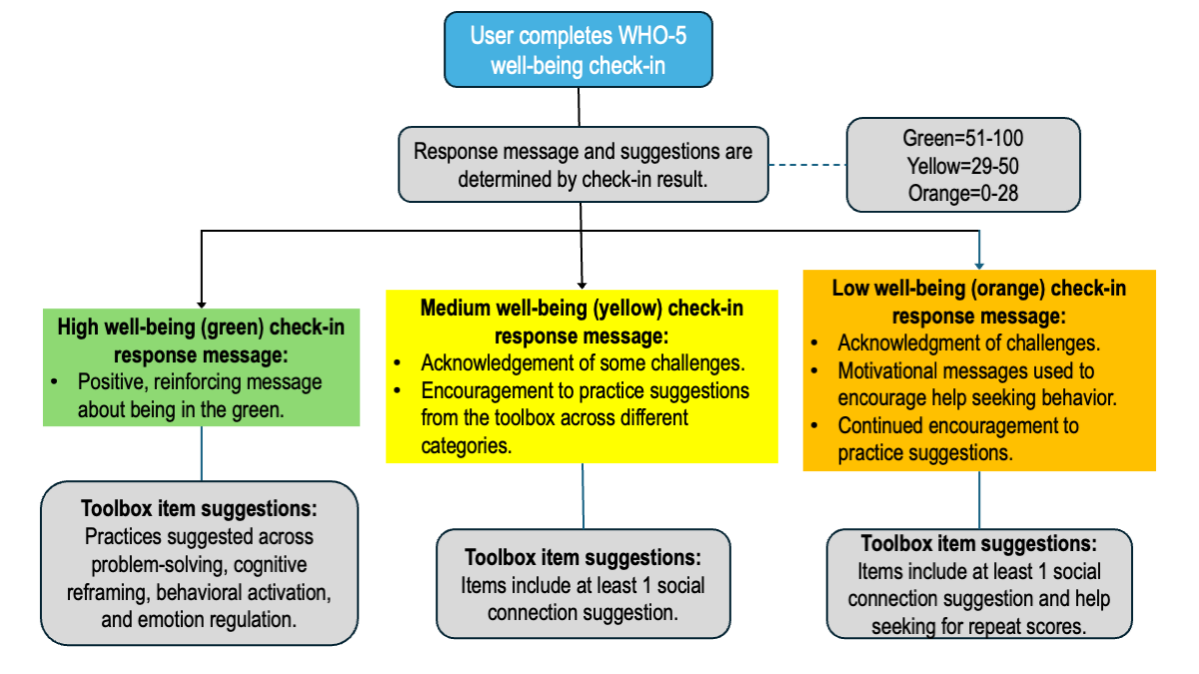
World Health Organization–Five Well-Being Index (WHO-5) well-being check-in responses.

In addition, the app uses a peer-based (*user-user*) approach, which identifies users with similar engagement patterns and recommends resources that those similar users have found helpful but the current user has not yet accessed. A second layer of intelligence involves user classification, where youth responses to in-app wellness screeners trigger tailored protocols and personalized content recommendations based on their identified needs. Specifically, Connected for Wellness includes the World Health Organization–Five Well-Being Index (WHO-5) [30], a short questionnaire that measures a person’s well-being over the previous 2 weeks and is suitable for children aged ≥9 years. Users can access this questionnaire on their own and will be reminded to complete it monthly through a notification. Higher scores on the WHO-5 indicate better mental well-being, whereas lower scores indicate potential risk of mental health concerns. After responding to the well-being questions, users receive a well-being score categorized as low, moderate, or high. On the basis of their score, they are provided with personalized well-being activities and suggestions, which can be saved in the app’s Toolbox section for easy access later. For example, a user who scores low on the WHO-5 will receive recommendations for professional help seeking, along with evidence-based strategies to support well-being, such as behavioral activation and emotion regulation techniques. Figure 1 illustrates how the WHO-5 normed scoring system guides the Toolbox recommendations and suggestions for help seeking provided to users based on their check-in results (ie, scores on the WHO-5: green** **for high well-being,** **yellow for moderate well-being, and orange for low well-being).

The primary objective of this study is to evaluate the effectiveness of the Connected for Wellness mobile app in enhancing youth engagement with mental health services when implemented in schools and primary care settings. This study aims to leverage the app’s ability to deliver evidence-based, culturally and contextually relevant information to address key barriers to mental health care, including stigma, limited accessibility, and the need for youth autonomy and confidentiality.

In addition, this study will explore how the app—co-designed with input from both youth and caregivers—enhances engagement with mental health support services and fosters help-seeking behaviors among youth and their families. Our main hypothesis is that the Connected for Wellness app will improve cascade-of-care outcomes compared to usual care. Specifically, we expect that youth at sites implementing the intervention will seek help more often, be screened more frequently, and be more likely to attend at least 3 mental health sessions if identified as needing services. If effective, the app has the potential to be a scalable tool for supporting youth mental health in community-based settings such as schools and primary care clinics and advancing public health approaches to connecting youth with care.

**Table 1:** Features of the Connected for Wellness mobile app for youth and caregivers.

Feature (English and Spanish)	Description	Examples
Pathways 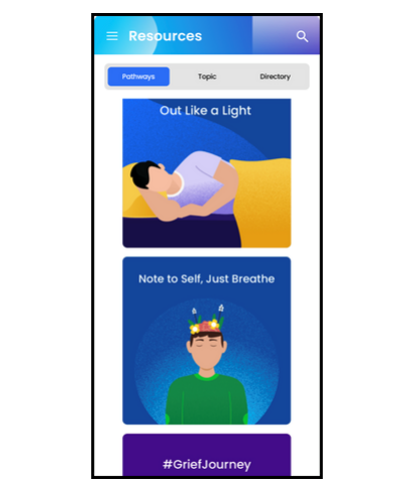	Brief videos and quick tips on well-being topics	Getting better sleep Improving communication Grief and loss Social connectedness Relaxation Managing emotions Academic stress Mental health help seeking
Self-assessments 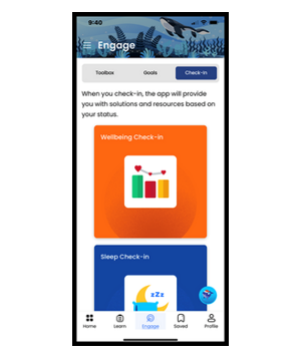	Short questionnaires to gauge sleep and well-being (for youth and caregivers) and social determinant of health needs (for caregivers)	WHO-5^a^ PROMIS^b^ sleep items Social determinant of health questions
Directory 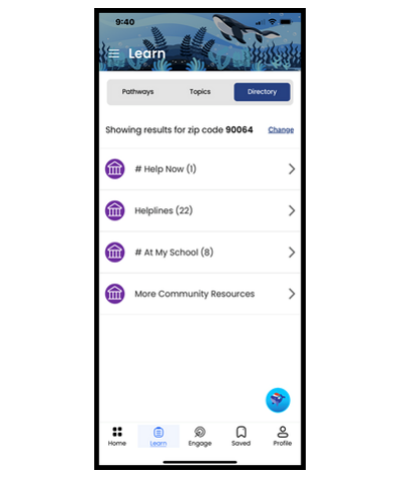	Site-specific, local, and national resources with direct dial, maps, and information about resources	Hotlines Helplines Clinic- or school-specific mental health and social resources located on-site Community resources (FindHelp)
Toolbox 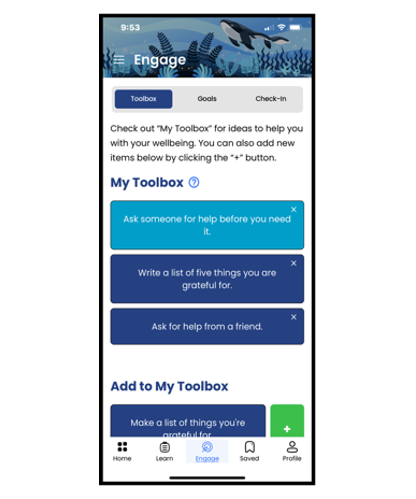	Over 500 well-being activities and suggestions that can be saved in the “Toolbox”	Brief suggestions based on well-being frameworks that include domains of physical, psychological, emotional, spiritual, relational, professional, and cultural well-being

^a^WHO-5: World Health Organization–Five Well-Being Index.

^b^PROMIS: Patient-Reported Outcomes Measurement Information System.

## Methods

### Objectives

This study protocol was developed in response to the National Institutes of Health, and the study was funded in August 2023 by the National Institute of Mental Health and will be completed by June 2027. In this section, we present the protocol for implementation of the mobile app using a stepped-wedge design in 10 schools and 10 clinics.

### Stepped-Wedge Design

We use a stepped-wedge design, a type of cluster randomized trial that involves clusters (such as groups of clinics or schools) randomly assigned to different time points (ie, waves) to begin receiving the intervention [31]. The rationale for the stepped-wedge design includes its effectiveness in testing health care delivery models and related outcomes for systems of care, particularly when randomization at the individual level is not feasible [32]. A stepped-wedge design is also ideal when an intervention must be rolled out gradually due to logistical, financial, or ethical constraints, ensuring that all groups eventually receive the intervention. It is particularly useful for evaluating complex, systemic, or behavioral interventions when simultaneous initiation of the intervention is not feasible. Because the goal is to offer the intervention to all youth and caregivers at participating study sites, 2 clusters—consisting of 5 schools and 5 primary care clinics per wave**—**are randomly assigned to 2 different implementation waves. All clusters begin in the control condition, with the Connected for Wellness mobile app intervention introduced to schools and clinics at predetermined time points. By the end of the trial, all clusters will have received the intervention, allowing for a stepped implementation across the sites. Stepped-wedge designs are becoming increasingly popular for testing health care delivery models [33] and are an innovative approach for evaluating the public health impact of mobile apps delivered as universal or tier 1 interventions in youth- and family-serving systems of care [34].

### Setting and Participants

This study will include youth aged 13 to 22 years (to include the range of possible ages in high school) and will be implemented across 10 public high schools and 10 community-based primary care clinics in 2 regions in California, United States. Caregivers will also be invited to use a version of the app for support of their child and their own well-being; however, this study does not monitor caregiver outcomes. Study sites will include urban and rural regions. The investigators will collaborate with one large school district and a network of primary care clinics that are part of a Federally Qualified Health Center system serving desert and rural regions. The schools and primary care clinics have existing peer support leaders or family navigators, who will integrate the Connected for Wellness app into their usual care in supporting students and caregivers in accessing mental health services and resources, encouraging help seeking, and using the in-app wellness skills.

### Recruitment Strategy and Site Selection

Schools and clinics were selected based on (1) geographic (urban and rural) and full demographic representation, (2) service to communities with high proportions of underserved youth (including low-income White, Latino, Black, and Asian backgrounds), and (3) existing collaborations with the investigative team. Sites had to demonstrate adequate infrastructure, including wellness navigators, school-based health centers, or peer educator programs. Recruitment within each site will involve promotional campaigns, informative sessions, videos, and peer navigator outreach.

### Existing Peer-to-Peer and Navigator Models

The high schools partnering with this study have an existing peer-to-peer model in which high school students are trained in a *community health framework* to deliver health and mental health education on topics relevant to their adolescent peers. This model targets health disparities by promoting wellness and reducing barriers to care within a culturally appropriate and community-centered approach [35,36]. Trained students lead school-wide campaigns to promote awareness and education with support from adult allies. In addition, each school has wellness navigators who will play a key role in integrating the mobile app into both school-wide educational efforts and individual support provided to students and caregivers. The primary care clinics participating in this study also have bilingual case managers and family navigators who will promote app use. These support specialists will be partnering with youth and families to help them access mental health information, develop wellness skills, and navigate linkages and referrals to care and community resources through the app.

### Participant Eligibility Recruitment, Consent, and Monitoring

For the study sites, 10 schools and 10 primary care clinics have been recruited to use the Connected for Wellness app site-wide, entering the study in 2 randomized cohorts (see Multimedia Appendix 1 for study timeline). All youth and caregivers at each intervention site will be offered the mobile app. Each site will have a site ID code to access the app. All youth aged 13 to 22 years at the site will be invited to participate in the study by using the mobile app. We will use an information kick-off event, other mental health and promotional events, website links, and videos that provide information about the study. Navigators, site champions, and peer ambassadors will also promote the app and help with downloads for participants requesting help (eg, caregivers who are less comfortable with technology). After all youth or caregivers download the app, they will choose their preferred language (English or Spanish) and role (student or caregiver).

As approved by the institutional review board (IRB) for this study, participants will then complete the in-app consent process, which presents study information in both video and text formats along with comprehension questions to ensure that participants understand the consent process. Only those who answer the questions correctly and indicate their willingness to proceed will gain access to the app. To maximize anonymity—a priority identified during the codevelopment phase—users do not enter any personally identifying information (such as name, email, date of birth, or password). Investigators will regularly review anonymous app data and maintain an aggregated dashboard by site, including demographic characteristics of app users. The dashboard will also capture app use patterns. Using insights from enrollment and app use data, the research team will work closely with school and primary care clinic study champions to actively promote study participation and engagement across the sites.

### Primary Outcomes: Cascade-of-Care Framework

The primary study outcomes are based on the cascade-of-care framework that focuses on population-level health needs and tracks individuals across relevant stages of a health care continuum [37,38]. Originally developed by the Juvenile Justice–Translational Research on Interventions for Adolescents in the Legal System [39,40], the steps in the cascade of care are identification of a behavioral health need, referral to behavioral health care, initiation of services, and engagement with services (>3 visits). The cascade of care has been adapted for youth in foster care [41]; this version was used for this study. Data on each step of the cascade of care will be obtained from deidentified electronic medical records and administrative data at the individual youth level from each of the study sites. This approach, which uses established databases, will enable comparison of cascade-of-care outcomes after implementation to data that were collected before preimplementation years. As detailed in the analytic plan in the Data Analysis section, individual-level data will be aggregated to the site level to test the hypothesis that implementation of the Connected for Wellness mobile app intervention is associated with improvements in youth progression through the cascade of care compared to the (preintervention) control condition. Improvements in the cascade of care are an established public health outcome as they help identify gaps in care and assess the effectiveness of the mobile app intervention. At a population level, in schools or primary care clinics, examining the cascade can inform how well an intervention improves youth access, engagement, and outcomes for a specific population and whether the intervention effectively addresses their unique needs and disparities. Statistical power was determined relative to the current level of each of the cascade outcomes (identification of mental health need, match to services, and initiation of services) in the sites as reflected in administrative data. Power was calculated separately for each cascade outcome. With a power of 0.8, an α value of .05, and 2-tailed tests, the planned design and sample sizes enable detection of small differences between the intervention and control conditions in all the cascade outcomes (2%-10%). Table 2 shows the operational definitions for the cascade-of-care outcome variables, and Table 3 shows the definitions, data collection, and outcomes.

**Table 2:** Cascade-of-care model based on Connected for Wellness triage outcomes.

Step	Rate	Operational definition
Youth at site (step a)	(a)	Total number of youth enrolled at a high school campus or on a patient panel at the primary care clinic site
Need identified (step b)	(b/a)	Subset of total enrolled youth (step a) who complete mental health and social determinant of health needs identification at school or the primary care clinic
Matched to services (step c)	(c/b)	Subset of youth with needs (step b) who receive a referral
Initiated services (step d)	(d/c)	Subset of those referred to services (step c) who initiate services (eg, first treatment session)
Engaged in services (step e)	(e/d)	Subset of those who initiate services (step d) who have ongoing sessions (≥3 visits)

**Table 3:** Primary outcomes based on the cascade-of-care framework and covariates for generalized linear mixed model (GLMM) analysis.

Cascade step	Operational definition and data description	Covariates in GLMM
Need identified	Youth who completed self-assessments or were flagged by navigators	Age, gender, race, ethnicity, site type (school vs clinic), and urban or rural
Matched to services	Referral documented in site records or follow-up logs	All the aforementioned covariates+previous mental health history if available
Initiated services	Attended first mental health appointment	Same as the previous covariates
Engaged in services	≥3 treatment sessions attended	Same as the previous covariates+time since app onboarding

### Ethical Considerations

This study has been reviewed and approved by the IRB at the University of California, Riverside (protocol 30090). All study procedures involving human participants adhere to institutional and national ethical standards. Informed consent is obtained via the app using both video and text formats. Participants complete comprehension questions to ensure understanding. Only youth who correctly answer the questions and indicate consent may proceed. Parental consent is required for youth aged <18 years and per institutional policy. No personally identifying information is collected. Data are fully anonymized, and site-level dashboards track aggregate use only. App use is voluntary, and users remain anonymous throughout. Participants are not compensated for using the app, which is designed as a universal public health intervention. Site staff (peer ambassadors and navigators) may receive stipends through partner institutions. No images or materials in this study contain identifiable participant information.

### Data Monitoring Plan

A data safety monitoring board (DSMB) will oversee the study to ensure participant safety, data integrity, and protocol adherence. The DSMB will review cumulative data at scheduled intervals and advise on any modifications needed to protect participants. The study team will conduct routine internal data quality checks, with any protocol deviations or serious concerns reported promptly to the DSMB and IRB.

### Data Management Plan

Study data will be collected using HIPAA (Health Insurance Portability and Accountability Act)-compliant, encrypted platforms, with all records linked to unique participant IDs to ensure confidentiality. Data will be stored on secure, password-protected institutional servers with regular backups. Data management procedures—including entry, coding, cleaning, and storage—will follow standard operating procedures and be documented for transparency and reproducibility.

### Plan for Collection and Reporting of Harms

All adverse events will be systematically recorded and reported per IRB and National Institutes of Health guidelines. Study personnel will be trained to monitor and respond to participant distress or risk disclosures, with serious adverse events reported to the DSMB and IRB within 48 hours. The DSMB will review all safety reports and provide recommendations for continuation or modification of study procedures as needed.

### Data Access and Use

Access to identifiable data will be limited to authorized study staff approved by the IRB. All data use will comply with established data use agreements with the participating schools and clinics.

### Dissemination Plan

All dissemination materials—including publications, presentations, and community reports—will be reviewed to ensure participant confidentiality and adherence to ethical standards. Community-facing materials will be developed in accessible formats and, when appropriate, cocreated with community partners.

### Data Analysis

Analyses will be conducted using the SAS statistical software (version 9.4; SAS Institute) [42]. Preliminary analyses will include examination of descriptive statistics, distributions, and internal consistency reliability of scaled measures. The primary hypotheses are that the Connected for Wellness mobile app will improve all cascade outcomes relative to the usual care control condition. Specifically, we hypothesize that the mobile app will affect site-level increases in the progression through the defined cascade of care, as defined in Table 2 (eg, youth at sites implementing the Connected for Wellness mobile app intervention will engage in more help seeking and be screened more frequently, and once screened and identified as needing mental health services, youth will be more likely to attend at least 3 sessions as compared to the control conditions [before implementation of the mobile app intervention]).

These hypotheses will be tested within a generalized linear mixed model (GLMM) framework implemented using the SAS PROC GLIMMIX statement. These models accommodate variously distributed dependent variables, including dichotomous outcomes such as those reflecting the cascade-of-care outcomes, and account for correlations among measurements of the same participants and nesting of youth within sites. Models will use a binomial distribution and a logit link function. All tests will be 2-tailed with a significance level of *P*=.05. For dichotomous outcomes, we will use a binomial distribution with a logit link function.

The GLMM models will include county and intervention (vs control) as fixed effects, with site and youth as random effects. Study month will be treated as a continuous variable to account for potential secular trends. The primary focus will be on the intervention effect. For youth with mental health need, we will also examine time-to-event outcomes (eg, days to service initiation) using Poisson models with a log link function. If overdispersion is detected, we will use a negative binomial model. The relative risk ratio for intervention will indicate whether the app reduces time to service matching and initiation. To address heterogeneity, the GLMM will include random intercepts for sites and fixed effects for county and wave. We will test for interactions between intervention status and site characteristics (eg, urban vs rural and school vs clinic). We will also conduct stratified analyses by key demographic variables (race and ethnicity, age, and gender) to explore subgroup effects.

## Results

As of May 2025, preparatory activities have been completed. These include finalization of the Connected for Wellness mobile app, IRB approvals, staff training, and recruitment of 20 participating sites (10 schools and 10 clinics randomized to cohorts). App deployment and participant recruitment are expected to begin between August 2025 and September 2025. App data collection and analysis of both app data and administrative data from the sites will begin in early 2026. Preliminary analyses are anticipated for October 2026. No results related to the study hypotheses are available at this time, but baseline engagement metrics and user demographics will be summarized once data are available. IRB approval was granted on July 1, 2025.

## Discussion

### Expected Findings

Innovative aspects of our clinical trial include the use of a mobile app codeveloped through participatory informatics integrating youth and caregiver priorities into its design to ensure that it addresses their specific needs. The app incorporates machine learning–driven, customizable features that offer personalized mental health resources focusing not only on mental health but also on addressing social determinants of health, such as housing and educational needs. By promoting local resources and providing tailored, real-time suggestions, the app helps bridge gaps in both mental health care and social services, ensuring that youth and caregivers can access the support they need in their everyday environments.

The growing body of evidence on mobile apps for mental health highlights their potential to improve access to care, engagement, and outcomes, particularly for underserved populations. While mobile health app technologies are increasingly recognized as scalable and accessible tools, their effectiveness is often dependent on personalization and relevance to the user’s context. Few studies have focused on participatory, co-design approaches tailored for youth and caregivers, which is where our study stands apart. By integrating machine learning, the app can dynamically adjust to the needs of each user, positioning it as an innovative solution in the field of mobile health.

Low-income populations, who often face barriers to accessing traditional health care [2], demonstrate high levels of cell phone and mobile internet use [43]. This provides a unique opportunity for mobile apps to address health care disparities. Research has shown that emergency departments increasingly serve as safety nets for underserved populations, especially for low-acuity conditions that could be managed in primary care settings. Mobile app tools designed to connect individuals with timely medical advice could reduce emergency department use, improving overall health care access [44]. While the potential exists, understanding the feasibility of underserved populations engaging with mobile apps to improve mental health care is essential [45]. A recent review of 36 trials conducted among youth found that, while mobile apps show promise in addressing issues such as depression, anxiety, and substance use, the evidence remains uncertain or limited [46]. Most of the studies included in the aforementioned review were small in scale, focused primarily on efficacy for youth already identified as at risk of depressive symptoms, and highlighted the need for larger trials to assess effectiveness and explore subgroup differences. Our study aims to address these gaps in scientific literature by testing a comprehensive, participatory, and machine learning–driven mobile app designed to improve wellness practices as a form of prevention while also improving service use outcomes for youth. This study places a strong emphasis on population health, embedding the intervention within school and primary care clinic settings.

### Limitations

The stepped-wedge design offers several advantages, including the ability to evaluate the intervention’s effectiveness while ensuring that all students in a high school or primary care clinic have the opportunity to access the wellness and prevention components of the app along with peer and family supports. It also allows for the assessment of temporal trends and the potential for learning or adaptation over time. However, this design also has limitations. The sequential implementation of the intervention may introduce biases such as those arising from differences in timing or external events affecting specific sites. Although the mobile app helps ensure fidelity by providing a standardized intervention across sites, the design still requires careful planning to manage logistical complexities and ensure consistency in implementation across diverse settings. Finally, variability in site readiness and capacity to implement the intervention highlights the need for robust support and ongoing monitoring of study sites. This includes tracking factors such as staff size, mental health service capacity, and the presence of existing peer-to-peer programs throughout the study.

### Conclusions

If successful, this study will demonstrate how mobile apps can serve as universal mental health and prevention tools, complementing existing behavioral health resources and community strategies such as peer support and family navigation programs. The mobile app–supported public health approach offers an equitable, scalable solution that has the potential reach and effectiveness to significantly improve public health outcomes and could reduce mental health care stigma among underserved communities.

## Appendix

Multimedia Appendix 1 Project Timeline for Stepped-wedge trial of the Connected for Wellness mobile app.

Multimedia Appendix 2 Peer-review report by ZRG1 MOSS-T (50) Center for Scientific Review Special Emphasis Panel RFA-RM-21-021: UNITE Transformative Research to Address Health Disparities and Advance Health Equity (U01), National Institute of Mental Health (National Institutes of Health, USA).

## Abbreviations

DSMB: data safety monitoring board
GLMM: generalized linear mixed model
HIPAA: Health Insurance Portability and Accountability Act
IRB: institutional review board
WHO-5: World Health Organization–Five Well-Being Index

## Data Availability

The datasets generated or analyzed during this study are not publicly available because they include administrative data owned by partnering schools and primary care sites, both of which are obligated to protect the privacy of minors.
