# Supplementary figures and images for: Addressing Youth Mental Health Through Schools and Primary Care Clinics Using the Connected for Wellness Mobile App: Protocol for a Stepped-Wedge Trial
Source: JMIR Res Protoc. 2025 Aug 26;14:e73721. doi: 10.2196/73721 (PMC12421200; doi:10.2196/73721)

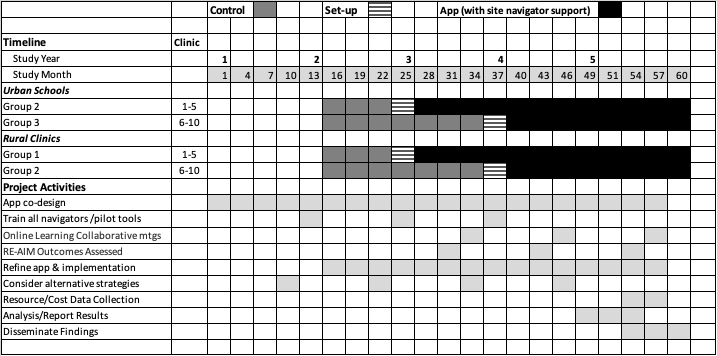


**Project Timeline for Stepped-wedge trial of the Connected for Wellness mobile app**

Supplement: Multimedia Appendix 1 [file resprot_v14i1e73721_app1.docx]
