# Supplementary material for: Addressing Youth Mental Health Through Schools and Primary Care Clinics Using the Connected for Wellness Mobile App: Protocol for a Stepped-Wedge Trial
Source: JMIR Res Protoc. 2025 Aug 26;14:e73721. doi: 10.2196/73721 (PMC12421200; doi:10.2196/73721)
Supplement: Multimedia Appendix 2 [file resprot_v14i1e73721_app2.pdf]

1U01DE031637-01 FORTUNA, LISA

RESUME AND SUMMARY OF DISCUSSION: The goal of this application is to develop a model of care for children and young adults to improve connections and access to mental health services and social support. Reviewers agreed that African American and Latino youth have up to three times greater odds of experiencing an unmet mental health need compared to their white counterparts and are more likely to be negatively impacted by social determinants of mental health related to poverty. Reviewers acknowledged applicants' novel idea to build an open access innovative technology combined with mental health navigation model to ameliorate mental health disparities for youth of color. It was felt that Youth and family navigators may improve connections to mental health and social services, and if successful, the project will provide novel information on how to best implement technology and navigation as scalable strategies for addressing disparities in access to mental health. Reviewers acknowledged a strong team of investigators that have the needed expertise to conduct the project. The team is experienced in the use of a community-based participatory research design and increases reviewers' confidence to implement the intervention successfully. The applicants have developed rigorously designed approaches with the community's engagement and inclusion of the participants in developing the app. It was expected that the app will reduce accessibility issues. Other strengths include solid rigor of the prior research, strong community/collaborative involvement, and the use of the Cascade of Care model. The project is highly scalable and was considered to have high transformative potential. However, during the discussion reviewers also raised few concerns about the novelty of the technology, feasibility issues due to several sites being studied, and a wide range of age groups of children involved in the development of the App. Few reviewers felt that the study design is geared towards implementation science without establishing the efficacy of the intervention. Based on several strengths and few minor weaknesses, reviewers viewed this project to elicit an impact on the mental health outcomes of the youth of color and the study is scalable for a sustainable uptake of the intervention. Thus, the overall enthusiasm of the review panel remained moderate for this application.

DESCRIPTION (provided by applicant): Despite significant progress in research, practice, and policy over the past few decades, many children and youth continue to experience poor mental health outcomes based on their socioeconomic disadvantage, ethnic or racial minority status, or immigrant status. African American and Latino youth have 1.5–3 times greater odds of experiencing an unmet mental health need than do their white counterparts and are more likely to be negatively impacted by social determinants of mental health related to poverty. With their unrivaled ability to reach youth, school-based and pediatric primary care services are ideal hubs to provide mental health, healthcare, social services, and prevention to students and families who otherwise face barriers to care. Using Participatory Design and Community Partnered Participatory Research (CPPR), UCLA and UCSF psychiatry research centers with Los Angeles Trust for Children's Health and San Francisco Health Network propose to: (1) Fully co-design (with youth, caregivers, clinicians and other stakeholders) an innovative mental health digital tool, called 4Youth, to implement algorithmically supported mental health and social determinants screening and triage, resiliency apps and navigation activities AND help support the primary care-clinical workforce within school centers and pediatric services; (2) Study the implementation of two mental health navigation models separately (family navigaton+4Youth and youth navigation+4Youth), and their combined effectiveness for improving connecting and matching youth to the right level of care and supports. This project will be initiated with youth 11-24 years old and family and community members across 10 Los Angeles Unified School District (LAUSD) Wellness Centers and 10 San Francisco Health Network pediatric primary care centers, which serve mostly Black, Latino, and Asian children. Mobile technology approaches are gaining empirical support and hold great potential for enhancing mental health navigator models. Incorporating scalable digital health tools, and statistically evaluated algorithms to aid the navigation process, such as screening, triage, tracking, connecting to care, and multi-level communication, will help ensure youth are receiving optimal care that navigators, providers and other relevant systems can measure. A successful outcome of the

project is a CPPR developed open-source intervention implementable in school-based and pediatric primary care services, for improving mental health services access for minoritized youth.

PUBLIC HEALTH RELEVANCE: African American and Latino youth are more likely to experience an unmet mental health or psychosocial need than do their white counterparts. School-based and pediatric primary care services are ideal hubs to provide mental health, healthcare, social services, and prevention to students and families who otherwise face barriers to care. Using Participatory Design and Community Partnered Participatory Research (CPPR), the proposed intervention is a model of care for connecting youth ages 11-24 to mental health care and supports by combining mobile technology for screening, triage and care coordination, co-created with the community and used by culturally responsive individuals called family and youth navigators, in school-based and pediatric primary care services.

CRITIQUE 1

Significance: 3
Investigator(s): 2
Innovation: 3
Approach: 4
Environment: 1

Overall Impact: This well-written proposal focuses on the unmet mental health needs of BIPOC youth. Efficient and universal screening processes, which also include social determinants of health and family adverse experiences, are needed to match youth needs to the right type of care. A multidisciplinary team across the UCSF and UCLA campuses, in collaboration with the LA Trust for Children's Health (which oversees school wellness centers in LAUSD) and the SF Health Network, aims to develop a mental health screening tool/navigator app created through participatory informatics to address disparities in access to mental health services by minoritized youth. They propose to use Community Partnered Participatory Research (CPRR) informatics to co-design a mental health digital tool (called 4Youth), integrate a mental health screening measure into this tool driven by iterative statistical analysis to identify youth risks, mental health and social needs with increasing precision, and use a stepped wedge design to test implementation of 4Youth with supported mental health navigation models (i.e., youth navigators and family navigators) to improve connections and access to mental health services and social support and reduce stigma associated with mental health care. If successful, the project would inform how to best implement technology and navigation as scalable strategies for addressing mental health access disparities. The proposal has strengths and transformative potential in attempting to address important barriers to care with transformative potential (improved screening, matching minoritized youth to the right level of care with existing available interventions, and decreasing stigma through peer/family navigation, and innovative in the methods proposed to develop the app). The team is well-positioned to do this work and there is clear emphasis on community engagement in developing the app and in roles as patient navigators. Mild to moderate weaknesses (i.e., the evidence-base for the efficacy of the interventions that they are proposing appears limited); it is not clear how the developmental needs across the large age continuum will be addressed, these decrease enthusiasm for an otherwise strong proposal.

1. Significance:

Strengths

- African American and Latino youth have much greater odds of experiencing an unmet mental health need than their white counterparts. There are numerous barriers to care (long wait times)

and youth perceptions (stigma, embarrassment, lack of awareness about services available or lack of awareness that one needs for care).

- Primary care and school wellness centers are the primary avenue through which mental health needs are addressed for publicly insured children but subject to long waiting lists and triaging for even one student can take 2-10 hours which diminishes resources available for direct mental health services. There is a need for targeted care coordination and navigation to increase linkages to mental health and community resources from primary care and school-based services.
- A mental health navigator app created through participatory informatics may help to address disparities in access to mental health services by minoritized youth. Youth navigator and/or family navigator (trained community staff who provide family support). There is limited research integrating navigators into pediatric services or school settings to address mental health but this has been done with social determinants of health in primary care, decreasing social needs and improving child health. Youth and family navigators may improve connections to mental health and social services and reduce stigma.
- If successful the project will inform how to best implement technology and navigation as scalable strategies for addressing mental health access disparities.

Weaknesses

- From their synopsis of the literature, the evidence basis for the interventions that they are proposing is limited. They briefly describe a recent pilot study of the app and mental health navigator program which found 50% of youth linked to community mental health services, compared to 20% of youth receiving standard of care. However, there is not enough information about the methods used/sampling procedure etc. from this brief description to determine how meaningful this difference is and gauge the potential efficacy of the intervention.

2. Investigator(s):

Strengths

- The PI, Dr. Fortuna at Child of psychiatry at UCSF Zuckerberg San Francisco General Hospital is child and adolescent psychiatrist and health services researcher with expertise in post-traumatic stress disorder (PTSD), depression and implementation research for improving access to mental health services by traditional underserved populations and through integrated behavioral health services in primary care. PI is on national and international NIMH, NIDA, and foundation-funded studies of innovative models and digital interventions, mental health care disparities in adult and pediatric populations, implementation research and pragmatic trials in primary care setting. Current MHR01 MPI optimizing family navigator model of child mental health. Dr. Kataoka – also child psychiatrist and health services researcher – emeritus professor at UCLA – involved in improving access to and quality of mental health care for ethnic minority youth and families in public settings such as schools. Brings a CBPR framework to the research. She has experience as site PI – SAMHSA and state level mechanisms.
- CO-Is: Dr. Porche (UCSF): Developmental psychologist, associate professor at UCSF. Works has focused on immigrant children and families building relationships with stakeholders from local refugee communities and co-designing family interventions through learning collaborative meetings. Dr. Folk (UCSF) clinical psychologist – has NICA K23 career training grant to develop and study technology-based interventions to improve outcomes for underserved youth and families Dr. Del Cid: transitioning to assistant professor in October 2021 – licensed bilingual bicultural clinical psychologist – overseeing navigation protocols in the project – she has served as a family navigator on a family mental health navigation project in pediatric primary care Dr. Shumway: Professor UCSF Psychiatry methods and statistics expert – cost outcome methods

for mental health – research methods for building evidence for intervention effectiveness in underserved community settings. Dr. Ijadi-Maghsoodi – child psychiatrist and health services researcher - assistant professor - focused on improving care for under-resourced youth and families through school, health centers, and community partnered mixed methods approaches. Leading stakeholder groups

- Consultant: Armen Arevian – at Chorus Innovations to contribute tech design, user experience research, community engagement to help the team implement the technology platform and advise on participatory development process.
- Community partners consultants (Sheryl Davis and Nicole Elmore) who have partnered with Dr. Fortuna on a community event focused on mental health and communities of color – from stigma to solutions. Community partners as co-investigators are strengths.

Weaknesses

- No LOS provided from community partner consultants re: their role on or support of the project.

3. Innovation:

Strengths

- In developing/refining the 4Youth App, the research team will use CPPR to drive technological innovation and take into account youth and family priorities in the development of the app, claiming that it will be the first CPPR designed technology and navigation program for minoritized youth.
- Pairing the app with peer and family navigators also increases the innovation of the proposal. If successful, this combination would be easily scalable.

Weaknesses

- App-based mental health screening tools combined with patient navigators are not particularly novel.

4. Approach:

Strengths

- CPPR and participatory design, emphasizing youth family and community voices in digital intervention development.
- Social networks theory analysis to show how technology supported navigation strategies influence change for the individual child and family and influence connectedness of resources and agencies within a multi-level, multi-sector approach and cascade of care.
- Access and analyze administrative databases across Wellness Centers and public health systems to build an initial predictive model for triage. Algorithm model pools constructs that also tap strength-based indicators.
- Triage based on screening tool into mild distress, moderate, or severe, linked to appropriate level of care.
- Primary outcomes are tangible and pragmatic: screening rates, triage to tiered care, and initiation of care at 12 months.
- Stepped wedge design for implementing a manualized family navigation model and manualized youth navigation model.
- Analysis plan will explore differences associated with biological sex, gender, race, ethnicity, and age.

Weaknesses

- Wide age range of participants (11-24) will likely make it difficult to design materials that are appealing/engaging and effective at screening across this continuum. How will youth navigators work with young participants? More information about how the intervention will meet the needs across this vast developmental continuum is needed.
- Study design is geared towards implementation science but efficacy of the intervention(s) does not appear to be established.

5. Environment:

Strengths

- The requisite expertise and resources are available at the participating institutions and partner sites. Strong letters of support from partners at LA Trust and SF Department of Health Director.

Weaknesses

- None noted by reviewer.

Study Timeline:

Strengths

- Timeline illustrates stepped wedge design and study activities; includes 6-month buffer period at the end; they describe strategies for entering more clinics sooner depending on progress.

Weaknesses

- None noted by the reviewer.

Protections for Human Subjects

Acceptable Risks and/or Adequate Protections

- Plan reasonable to identify and mitigate risk provided

Data and Safety Monitoring Plan (Applicable for Clinical Trials Only):

Acceptable

- o DSMB and description of reporting UAE and EAE identified.

Inclusion Plans

- Sex/Gender: Distribution justified scientifically
- Race/Ethnicity: Distribution justified scientifically
- For NIH-Defined Phase III trials, Plans for valid design and analysis: Scientifically acceptable
- Inclusion/Exclusion Based on Age: Distribution justified scientifically

Vertebrate Animals

Not Applicable (No Vertebrate Animals)

Biohazards

Not Applicable (No Biohazards)

Select Agents

Not Applicable (No Select Agents)

Resource Sharing Plans

Acceptable

Authentication of Key Biological and/or Chemical Resources

Not Applicable (No Relevant Resources)

Budget and Period of Support

Recommend as Requested

CRITIQUE 2

Significance: 2
Investigator(s): 1
Innovation: 1
Approach: 2
Environment: 1

Overall Impact: This is an exceptionally strong project that addresses a problem of high importance and covers multiple areas of concern based on the FOA. It builds on prior work of high standards and will have the capacity to inform the best methods for implementing technology and navigation as scalable strategies for addressing access in mental health disparities and diminishing mental health care stigma in minoritized youth. It generates excitement in that it is first CPPR designed technology and mental health navigation program with a high potential of improving disparities in access to care in a timely, well matched manner in two major systems of care.

1. Significance:

Strengths

- This study is responsive to the FOA in multiple ways: 1) it uses Participatory Design and Community Partnered Participatory Research (CPPR) as the basis from which all other work will be conducted, 2) it is technically innovative and 3) it will impact two major child serving systems of care and, 4) it has the potential to be a scalable intervention for children and adolescents who have struggled through the COVID-19 pandemic and will need support beyond post-pandemic times.
- The team demonstrated exceptional expertise and readiness to implement “By Youth, For Youth”.
- The Cascade of Care Model and the RE-AIM framework are well adapted to fit the primary aim; to address the disproportionate poor access to mental health services by minority youth and families.
- This study is positioned to lead to a sustainable uptake of the intervention and with improved mental health for youths across other systems of care with the added benefit of helping support the primary care-clinician workforce within school centers and pediatric services.

Weaknesses

- Engaging clinicians to use the digital platform may be one of the more challenging aspects of the project.

2. Investigator(s):

Strengths

- The research team is outstanding and has a long-standing history of collaboration.
- The multidisciplinary team has extensive experience in the fields of trauma, mental health, CPPR, behavioral health interventions and intervention research, family navigator models, and digital health services research, among others.
- Both co-PIs are committing adequate effort to the project (20%/15%).

Weaknesses

- None noted by the reviewer.

3. Innovation:

Strengths

- This would be the first study of its kind and the benefits, if successful, are far reaching.
- Out of all components the one that stands out is the 4Youth mental health navigator app created through participatory informatics to address disparities in access to mental health care.
- The use of CPPR as a principal that leads all stages of the project in innovative ways as previously mentioned.

Weaknesses

- None noted by the reviewer.

4. Approach:

Strengths

- The proposed project demonstrates a solid understanding of the difficulties confronted by the population of interest in terms of unmet need and the higher odds of being negatively impacted by social determinants of mental health related to poverty.
- The team takes advantage of prior experience in this field that are needed to implement the intervention. The project is well supported by preliminary data.
- The connections that have already been established with LA Trust for Children's Health and San Francisco Health Network can provide the team with the possibility of a rapid implementation of the intervention.
- The team is experienced in the use of a community-based participatory research design. The team's leadership and management are appropriate and has the capacity to engage the population needed to achieve the aims of the proposal.
- The implementation of the current study comes at a time that especially meaningful due to the disparities and challenges that have been exacerbated in the wake of COVID-19.
- The strategies presented ensure a robust and unbiased approach.

- Chorus has multi-language capabilities, and the technology co-development groups will include Spanish-speaking bilingual members.
- The study design is justified and well described. The underlying logic or rationale for each strategy is discussed.
- Digital collection through the 4Youth app and the use of qualitative survey and interviews will allow a mix-methods evaluation of effectiveness of technology supported navigation.
- Takes advantage of existing collaborations to implement study design.
- *Data Management and Statistical Analysis:* This study has a well-designed data management and analysis plan.
- The primary analyses for which cascade outcomes are the dependent variable contemplate being able to code specific time periods and examine their impact (i.e., COVID 19 pandemic).

Weaknesses

- Study should contemplate the possibility of other interventions in the same school district and how it will impact the current proposal.
- Under alternate approaches that may be pursued, unclear as to the data that will be collected via stakeholder meetings twice a year.

5. Environment:

Strengths

- The environment at both sites is outstanding and the team has all the support needed to successfully implement the project.

Weaknesses

- None noted by the reviewer.

Study Timeline:

Strengths

- The milestones for all stages of the project are provided and are appropriate.
- The stepped wedge design is clearly depicted. It considers intermediate outcomes and timepoints to implement alternative strategies.

Weaknesses

- None noted by the reviewer.

Protections for Human Subjects

Acceptable Risks and/or Adequate Protections

- Acceptable.

Data and Safety Monitoring Plan (Applicable for Clinical Trials Only):

Acceptable

Inclusion Plans

- Sex/Gender: Distribution justified scientifically

- Race/Ethnicity: Distribution justified scientifically
- For NIH-Defined Phase III trials, Plans for valid design and analysis: Not applicable
- Inclusion/Exclusion Based on Age: Distribution justified scientifically

Vertebrate Animals

Not Applicable (No Vertebrate Animals)

Biohazards

Not Applicable (No Biohazards)

Select Agents

Not Applicable (No Select Agents)

Resource Sharing Plans

Acceptable

Authentication of Key Biological and/or Chemical Resources

Not Applicable (No Relevant Resources)

Budget and Period of Support

Recommend as Requested

CRITIQUE 3

Significance: 1
Investigator(s): 1
Innovation: 2
Approach: 3
Environment: 1

Overall Impact: The goals of this project are to develop, using a Participatory Design and Community Partnered Participatory Research (CPPR) approach, a model of care, 4Youth, to connect children and young adults aged 11-24 years to mental health care and supports. The project will be implemented in school-based and pediatric primary care services. 4Youth is a mental health navigator app mobile technology for screening, triage and care coordination. Strengths include a strong investigative team that includes community organizations, use of strong conceptual and guiding framework for health care, and methodological approaches appropriate to the aims. Weaknesses involve a wide range of age groups and sites with different needs. However, these weaknesses should be offset by the proposed methodological approaches. The proposed approaches have a high likelihood of resulting in transformative changes.

1. Significance:

Strengths

- The proposed study addresses a significant issue – the unmet mental health needs of African American and Latino children and young adults.
- If the aims of the project are achieved, the project will produce a community-involved and developed open-source intervention that can be implemented in school-based and pediatric primary care services, for improving mental health services access for under-served youth.

Weaknesses

- None noted by reviewer.

2. Investigator(s):

Strengths

- There is a strong investigative team that is well suited to the project
- Importantly, the co-investigators include executive directors and other key members of community groups.

Weaknesses

- None noted by reviewer.

3. Innovation:

Strengths

- Youth navigators are novel as they are not commonly employed.

Weaknesses

- mHealth technology is not particularly innovative.

4. Approach:

Strengths

- There is strong community/collaborative involvement. The project will be fully co-designed with youth, caregivers, clinicians, and other stakeholders.
- The proposed plans to study the implementation of two mental health navigation models separately (family navigaton+4Youth and youth navigation+4Youth) are appropriate and are strengths.
- The stepped wedge design is a strength.
- Use of the Cascade of Care model is a strength.
- Rigor of the prior research that serves as the key support for the proposed project is well-described.
- There is a good discussion of challenges and alternative approaches.

Weaknesses

- The program targets wide age ranges that include various developmental stages and potentially different mental health challenges. These age groups include potentially different needs.
- Many different sites and clinics (in Los Angeles and San Francisco) are proposed for the study. These include 10 school districts. There is a strong likelihood that the needs of these sites will vary greatly.

- It is possible that having appropriate community involvement will help to address these issues. It would have been helpful to see a discussion of how the project will deal with these potential age and site differences.

5. Environment:

Strengths

- There is a strong research environment.
- The project will benefit from strong community involvement.

Weaknesses

- None noted by reviewer.

Study Timeline:

Strengths

- Timeline is appropriate.

Weaknesses

- None noted by reviewer.

Protections for Human Subjects

Acceptable Risks and/or Adequate Protections

Data and Safety Monitoring Plan (Applicable for Clinical Trials Only):

Acceptable

Inclusion Plans

- Sex/Gender: Distribution justified scientifically
- Race/Ethnicity: Distribution justified scientifically
- For NIH-Defined Phase III trials, Plans for valid design and analysis: Not applicable
- Inclusion/Exclusion Based on Age: Distribution justified scientifically

Vertebrate Animals

Not Applicable (No Vertebrate Animals)

Biohazards

Not Applicable (No Biohazards)

Select Agents

Not Applicable (No Select Agents)

Resource Sharing Plans

Acceptable

Authentication of Key Biological and/or Chemical Resources

Not Applicable (No Relevant Resources)

Budget and Period of Support

Recommend as Requested

THE FOLLOWING SECTIONS WERE PREPARED BY THE SCIENTIFIC REVIEW OFFICER TO SUMMARIZE THE OUTCOME OF DISCUSSIONS OF THE REVIEW COMMITTEE, OR REVIEWERS' WRITTEN CRITIQUES, ON THE FOLLOWING ISSUES:

PROTECTION OF HUMAN SUBJECTS: ACCEPTABLE

INCLUSION OF WOMEN PLAN: ACCEPTABLE

INCLUSION OF MINORITIES PLAN: ACCEPTABLE

INCLUSION ACROSS THE LIFESPAN: ACCEPTABLE

COMMITTEE BUDGET RECOMMENDATIONS: The budget was recommended as requested.

Footnotes for 1 U01 DE031637-01; PI Name: FORTUNA, LISA R

NIH has modified its policy regarding the receipt of resubmissions (amended applications).See Guide Notice NOT-OD-18-197 at https://grants.nih.gov/grants/guide/notice-files/NOT-OD-18-197.html. The impact/priority score is calculated after discussion of an application by averaging the overall scores (1-9) given by all voting reviewers on the committee and multiplying by 10. The criterion scores are submitted prior to the meeting by the individual reviewers assigned to an application, and are not discussed specifically at the review meeting or calculated into the overall impact score. Some applications also receive a percentile ranking. For details on the review process, see http://grants.nih.gov/grants/peer_review_process.htm#scoring.

MEETING ROSTER

Center for Scientific Review Special Emphasis Panel
CENTER FOR SCIENTIFIC REVIEW
RFA-RM-21-021: UNITE Transformative Research to Address Health Disparities and Advance Health Equity (U01)
ZRG1 MOSS-T (50)
07/27/2021 - 07/28/2021

Notice of NIH Policy to All Applicants: Meeting rosters are provided for information purposes only. Applicant investigators and institutional officials must not communicate directly with study section members about an application before or after the review. Failure to observe this policy will create a serious breach of integrity in the peer review process, and may lead to actions outlined in NOT-OD-14-073 at https://grants.nih.gov/grants/guide/notice-files/NOT-OD-14-073.html, NOT-OD-15-106 at https://grants.nih.gov/grants/guide/notice-files/NOT-OD-15-106.html, and NOT-OD-18-115 at https://grants.nih.gov/grants/guide/notice-files/NOT-OD-18-115.html, including removal of the application from immediate review.

CHAIRPERSON(S)

MURRY, VELMA MCBRIDE, PHD
LOIS AUTREY BETTS CHAIR AND JOE B WYATT
DISTINGUISHED UNIVERSITY PROFESSOR
DEPARTMENT OF HUMAN AND ORGANIZATIONAL
DEVELOPMENT
VANDERBILT UNIVERSITY
NASHVILLE, TN 37203

MEMBERS

ABRAIDO-LANZA, ANA F., PHD
PROFESSOR
VICE DEAN SOCIAL AND BEHAVIORAL SCIENCES
SCHOOL OF GLOBAL PUBLIC HEALTH
NEW YORK UNIVERSITY
NEW YORK, NY 10012

ADUNYAH, SAMUEL E, PHD
PROFESSOR AND CHAIRMAN
DEPARTMENT OF BIOCHEMISTRY, CANCER BIOLOGY
NEUROSCIENCE AND PHARMACOLOGY
SCHOOL OF MEDICINE
MEHARRY MEDICAL COLLEGE
NASHVILLE, TN 37208

ARORA, KAVITA SHAH, MD
ASSOCIATE PROFESSOR
METROHEALTH MEDICAL CENTER
CASE WESTERN RESERVE UNIVERSITY
CLEVELAND, OH 44109

BENTLEY-EDWARDS, KEISHA L., PHD
ASSOCIATE DIRECTOR OF RESEARCH, SAMUEL DUBOIS
COOK CENTER ON SOCIAL EQUITY
DUKE UNIVERSITY
DURHAM, NC 27708

BORREGO, MATTHEW, PHD
PROFESSOR
DEPARTMENT OF PHARMACY PRACTICE
AND ADMINISTRATIVE SCIENCES
COLLEGE OF PHARMACY
UNIVERSITY OF NEW MEXICO
ALBUQUERQUE, NM 87131

BRUCE, MARINO A, PHD
CLINICAL PROFESSOR OF BEHAVIORAL AND SOCIAL
SCIENCES
COLLEGE OF MEDICINE
UNIVERSITY OF HOUSTON
HOUSTON, TX 77004

BURKE, NANCY J., PHD
DEPARTMENT CHAIR AND PROFESSOR
DEPARTMENT OF PUBLIC HEALTH
SCHOOL OF SOCIAL SCIENCES AND HUMANITIES
UNIVERSITY OF CALIFORNIA, MERCED
MERCED, CA 95343

CASSIDY-BUSHROW, ANDREA E, PHD
ASSOCIATE SCIENTIST AND RESEARCH EPIDEMIOLOGIST
DEPARTMENT OF PUBLIC HEALTH SCIENCES
HENRY FORD HEALTH SYSTEM
DETROIT, MI 48202

CASTRO, EIDA MARIA, PSYD
ASSOCIATE PROFESSOR
DEPARTMENT OF PSYCHIATRY
MENTAL HEALTH DIVISION
SCHOOL OF BEHAVIORAL AND BRAIN SCIENCES
PONCE SCHOOL OF MEDICINE
PONCE, PR 00716

CHAKKALAKAL, ROSETTE J, MD
ASSOCIATE PROFESSOR
DIVISION OF GENERAL INTERNAL MEDICINE AND PUBLIC HEALTH
MEDICAL CENTER
VANDERBILT UNIVERSITY
NASHVILLE, TN 37235

CHATTERJI, PINKA, PHD
PROFESSOR
ECONOMICS DEPARTMENT
UNIVERSITY AT ALBANY
ALBANY, NY 12222

CHAVEZ, LIGIA M., PHD
ASSOCIATE PROFESSOR
BEHAVIORAL SCIENCES RESEARCH INSTITUTE
UNIVERSITY OF PUERTO RICO
RIO PIEDRAS, PR 00935

COHN, ELIZABETH GROSS, PHD
RUDIN CHAIR AND PROFESSOR OF COMMUNITY-ENGAGED RESEARCH
ASSOCIATE PROVOST FOR RESEARCH
HUNTER COLLEGE
CITY UNIVERSITY OF NEW YORK
NEW YORK, NY 10065

CUBBIN, CATHERINE, PHD
PROFESSOR AND ASSOCIATE DEAN FOR RESEARCH
STEVE HICKS SCHOOL OF SOCIAL WORK
UNIVERSITY OF TEXAS AT AUSTIN
AUSTIN, TX 78712

DALE, SANNISHA K., PHD
ASSOCIATE PROFESSOR
HEALTH DIVISION
DEPARTMENT OF PSYCHOLOGY
UNIVERSITY OF MIAMI CORAL GABLES
CORAL GABLES, FL 33146

DIAZ, VANESSA ASTRUD, MD
ASSISTANT PROFESSOR
DEPARTMENT OF FAMILY MEDICINE
MEDICAL UNIVERSITY OF SOUTH CAROLINA
CHARLESTON, SC 29425

EHRENTHAL, DEBORAH BETH, MD, MPH
PROFESSOR
DEPARTMENT OF OBSTETRICS & GYNECOLOGY AND POPULATION HEALTH SCIENCES
UNIVERSITY OF WISCONSIN
SCHOOL OF MEDICINE AND PUBLIC HEALTH
MADISON, WI 53726

ERINOSHO, TEMITOPE O, PHD
ASSOCIATE PROFESSOR
DEPARTMENT OF APPLIED HEALTH SCIENCES
SCHOOL OF PUBLIC HEALTH
INDIANA UNIVERSITY BLOOMINGTON
BLOOMINGTON, IN 27599

GAMAREL, KRISTINE E, PHD
JOHN G. SEARLE ASSISTANT PROFESSOR
DEPARTMENT OF HEALTH BEHAVIOR AND HEALTH EDUCATION
SCHOOL OF PUBLIC HEALTH
UNIVERSITY OF MICHIGAN
ANN ARBOR, MI 48109

GONZALEZ, CRISTINA M, MD
PROFESSOR
DEPARTMENT OF MEDICINE
MONTEFIORE MEDICAL CENTER
ALBERT EINSTEIN COLLEGE OF MEDICINE
BRONX, NY 10461

HALL, WILLIAM JAMES, PHD
ASSISTANT PROFESSOR
SCHOOL OF SOCIAL WORK
UNIVERSITY OF NORTH CAROLINA CHAPEL HILL
CHAPEL HILL, NC 27599

HICKEN, MARGARET TAKAKO, PHD
RESEARCH ASSOCIATE PROFESSOR
SURVEY RESEARCH CENTER
INSTITUTE FOR SOCIAL RESEARCH
UNIVERSITY OF MICHIGAN
ANN HARBOR, MI 48104

HIRSHFIELD, SABINA, PHD
PRINCIPAL RESEARCH SCIENTIST
DEPARTMENT OF MEDICINE
STAR PROGRAM
SUNY DOWNSTATE HEALTH SCIENCES UNIVERSITY
BROOKLYN, NY 11203

KATZ, MIRA L, PHD
PROFESSOR
DEPARTMENT OF HEALTH BEHAVIOR AND HEALTH PROMOTION
COLLEGE OF PUBLIC HEALTH
OHIO STATE UNIVERSITY
COLUMBUS, OH 43210

KIM, DANIEL, MD, DRPH
ASSOCIATE PROFESSOR
DEPARTMENT OF HEALTH SCIENCES
BOUVE COLLEGE OF HEALTH SCIENCES
NORTHEASTERN UNIVERSITY
BOSTON, MA 02115

KUNIN-BATSON, ALICIA S, PHD
ASSISTANT PROFESSOR
DEPARTMENT OF PEDIATRICS
UNIVERSITY OF MINNESOTA MEDICAL SCHOOL
MINNEAPOLIS, MN 55414

LEONE, LUCIA A, PHD
ASSOCIATE PROFESSOR
DEPARTMENT OF COMMUNITY HEALTH AND HEALTH
BEHAVIOR
SCHOOL OF PUBLIC HEALTH AND HEALTH PROFESSIONS
STATE UNIVERSITY OF NEW YORK AT BUFFALO
BUFFALO, NY 14214

LI, YUE, PHD
PROFESSOR OF PUBLIC HEALTH SCIENCES
DIRECTOR, HEALTH SERVICES RESEARCH & POLICY (HSRP)
DIVISION OF HEALTH POLICY AND OUTCOMES RESEARCH
DEPARTMENT OF PUBLIC HEALTH SCIENCES
UNIVERSITY OF ROCHESTER MEDICAL CENTER
ROCHESTER, NY 14642

MARTINEZ, MARIA ELENA, PHD
SAM M. WALTON ENDOWED CHAIR FOR CANCER
RESEARCH
PROFESSOR AND ASSOCIATE DIRECTOR
POPULATION SCIENCES, DISPARITIES
AND COMMUNITY ENGAGEMENT
UC SAN DIEGO MOORES CANCER CENTER
LA JOLLA, CA 92093

MARTINEZ, PRISCILLA, PHD
ASSOCIATE SCIENTIST
ALCOHOL RESEARCH GROUP
PUBLIC HEALTH INSTITUTE
EMERYVILLE, CA 94608

MCDONOUGH, IAN, PHD
ASSOCIATE PROFESSOR
DEPARTMENT OF PSYCHOLOGY
COLLEGE OF ARTS AND SCIENCES
UNIVERSITY OF ALABAMA, TUSCALOOSA
TUSCALOOSA, AL 35487

MITCHELL, SUZANNE E, MD
ASSOCIATE PROFESSOR
DEPARTMENT OF FAMILY MEDICINE
SCHOOL OF MEDICINE
BOSTON UNIVERSITY
BOSTON, MA 02118

MITSIADES, NICHOLAS, MD, PHD
ASSOCIATE PROFESSOR
MEDICINE-HEMATOLOGY AND ONCOLOGY
DEPARTMENT OF MOLECULAR AND CELLULAR BIOLOGY
COLLEGE OF MEDICINE
BAYLOR COLLEGE OF MEDICINE
HOUSTON, TX 77030

MOSKOWITZ, JUDITH T, PHD
PROFESSOR
DEPARTMENT OF MEDICAL SOCIAL SCIENCES
FEINBERG SCHOOL OF MEDICINE
NORTHWESTERN UNIVERSITY
CHICAGO, IL 60611

ODERO-MARAH, VALERIE, PHD
PROFESSOR & ASSISTANT DIRECTOR OF RESEARCH
DEPARTMENT OF BIOLOGICAL SCIENCES
CENTER FOR CANCER RESEARCH AND THERAPEUTIC
DEVELOPMENT
CLARK ATLANTA UNIVERSITY
ATLANTA, GA 30314

PRESS, VALERIE G, MD
ASSOCIATE PROFESSOR
DEPARTMENTS OF MEDICINE AND PEDIATRICS
UNIVERSITY OF CHICAGO
CHICAGO, IL 60637

RANGACHARI, PAVANI, PHD
PROFESSOR
DEPARTMENT OF INTERDISCIPLINARY HEALTH SCIENCES
DEPARTMENT OF FAMILY MEDICINE (MCG)
THE GRADUATE SCHOOL
AUGUSTA UNIVERSITY
AUGUSTA, GA 30912

RICKS-SANTI, LUISEL J., PHD
DIRECTOR
CANCER RESEARCH CENTER
HAMPTON UNIVERSITY
HAMPTON, VA 23668

ROTE, SUNSHINE MARIE, PHD
ASSOCIATE PROFESSOR
KENT SCHOOL OF SOCIAL WORK
UNIVERSITY OF LOUISVILLE
LOUISVILLE, KY 40292

SCHEIM, AYDEN I, PHD
ASSISTANT PROFESSOR
EPIDEMIOLOGY AND BIOSTATISTICS
SCHOOL OF PUBLIC HEALTH
DREXEL UNIVERSITY
PHILADELPHIA, PA 19104

SHARIFF-MARCO, SALMA, PHD
ASSOCIATE PROFESSOR
DEPARTMENT OF EPIDEMIOLOGY AND BIOSTATISTICS
HELEN DILLER FAMILY COMPREHENSIVE CANCER CENTER
GREATER BAY AREA CANCER REGISTRY (GBACR)
UNIVERSITY OF CALIFORNIA, SAN FRANCISCO
SAN FRANCISCO, CA 94158

SONIK, RAJAN ANTHONY, JD, PHD
DIRECTOR OF RESEARCH
ALTAMED HEALTH SERVICES CORPORATION
LOS ANGELES, CA 90040

TEHRANIFAR, PARISA, DPH
ASSOCIATE PROFESSOR
DEPARTMENT OF EPIDEMIOLOGY
MAILMAN SCHOOL OF PUBLIC HEALTH
COLUMBIA UNIVERSITY
NEW YORK, NY 10032

TOBIN, KARIN E, PHD
ASSOCIATE PROFESSOR
DEPARTMENT OF HEALTH, BEHAVIOR, AND SOCIETY
BLOOMBERG SCHOOL OF PUBLIC HEALTH
JOHNS HOPKINS UNIVERSITY
BALTIMORE, MD 21205

TULU, BENGISU, PHD
PROFESSOR
BUSINESS SCHOOL
WORCESTER POLYTECHNIC INSTITUTE
WORCESTER, MA 01609

VUPPUTURI, SUMA, PHD
SENIOR RESEARCH SCIENTIST
MID-ATLANTIC PERMANENTE RESEARCH INSTITUTE
KAISER PERMANENTE MID-ATLANTIC
ROCKVILLE, MD 20852

WANG, JUNLING NONE, PHD
PROFESSOR AND VICE CHAIR FOR RESEARCH
DEPARTMENT OF CLINICAL PHARMACY
AND TRANSLATIONAL SCIENCE
COLLEGE OF PHARMACY
UNIVERSITY OF TENNESSEE HEALTH SCIENCE CENTER
MEMPHIS, TN 38163

WESCOTT, SIOBHAN M, MD
PROFESSOR & DIRECTOR OF AMERICAN INDIAN HEALTH PROGRAM
COLLEGE OF PUBLIC HEALTH
UNIVERSITY OF NEBRASKA MEDICAL CENTER
GRAND FORKS, ND 58202

WHITT-GLOVER, MELICIA C, PHD
PRESIDENT AND CHIEF EXECUTIVE OFFICER
GRAMERCY RESEARCH GROUP, LLC
ADJUNCT ASSOCIATE PROFESSOR
WAKE FOREST SCHOOL OF MEDICINE
WINSTON-SALEM, NC 27106

WILLIAMS, DONNA L., DRPH
PROFESSOR
DEPARTMENT OF BEHAVIORAL HEALTH SCIENCES
LOUISIANA COMPREHENSIVE CANCER CONTROL PROGRAMS
HEALTH SCIENCE CENTER
LOUISIANA STATE UNIVERSITY, NEW ORLEANS
NEW ORLEANS, LA 70112

WILLIAMS, JONI STROM, MD
ASSOCIATE PROFESSOR
DEPARTMENT OF MEDICINE
DIVISION OF GENERAL INTERNAL MEDICINE
MEDICAL COLLEGE OF WISCONSIN
WAUWATOSA, WI 53226

SCIENTIFIC REVIEW OFFICER

BEHERA, ARUNA K, PHD
SCIENTIFIC REVIEW OFFICER
CENTER FOR SCIENTIFIC REVIEW
NATIONAL INSTITUTES OF HEALTH
BETHESDA, MD 20892

EXTRAMURAL SUPPORT ASSISTANT

ROBINSON, LYNDA K., BS
LEAD GRANTS TECHNICAL ASSISTANT
CENTER FOR SCIENTIFIC REVIEW
NATIONAL INSTITUTES OF HEALTH
BETHESDA, MD 20892

OTHER REVIEW STAFF

FLEMING, LIA CAROLINE, MPH
REVIEW ANALYST
CENTER FOR SCIENTIFIC REVIEW
NATIONAL INSTITUES OF HEALTH
BETHESDA, MD 20892

HONG, SEO YOUNG, MPH
REVIEW ANALYST
CENTER FOR SCIENTIFIC REVIEW
NATIONAL INSTITTUES OF HEALTH
BETHESDA, MD 20892

JAIN, ADITI, BS, MPH
REVIEW ANALYST
CENTER FOR SCIENTIFIC REVIEW
NATIONAL INSTITUTES OF HEALTH
BETHESDA, MD 20892

Consultants are required to absent themselves from the room during the review of any application if their presence would constitute or appear to constitute a conflict of interest.
